# Promoting Self-Care of Diabetic Foot Ulcers Through a Mobile Phone App: User-Centered Design and Evaluation

**DOI:** 10.2196/10105

**Published:** 2018-10-10

**Authors:** Bernd Ploderer, Ross Brown, Leonard Si Da Seng, Peter A Lazzarini, Jaap J van Netten

**Affiliations:** 1 School of Electrical Engineering and Computer Science Queensland University of Technology Brisbane, QLD Australia; 2 School of Clinical Sciences Queensland University of Technology Brisbane, QLD Australia; 3 Allied Health Research Collaborative Metro North Hospital and Health Service Brisbane, QLD Australia; 4 Amsterdam UMC, University of Amsterdam Department of Rehabilitation Amsterdam Movement Sciences Amsterdam Netherlands

**Keywords:** mobile apps, foot ulcer, diabetic, self-care (rehabilitation), therapeutic adherence and compliance, patient engagement, podiatry

## Abstract

**Background:**

Without effective self-care, people with diabetic foot ulcers (DFUs) are at risk of prolonged healing times, hospitalization, amputation, and reduced quality of life. Despite these consequences, adherence to DFU self-care remains low. New strategies are needed to engage people in the self-care of their DFUs.

**Objective:**

This study aimed to evaluate the usability and potential usefulness of a new mobile phone app to engage people with DFUs in self-care.

**Methods:**

We developed a new mobile phone app, MyFootCare, to engage people with DFUs through goals, progress monitoring, and reminders in self-care. Key features included novel visual analytics that automatically extract and monitor DFU size information from mobile phone photos of the foot. A functional prototype of MyFootCare was created and evaluated through a user-centered design process with 11 participants with DFUs. Data were collected through semistructured interviews discussing existing self-care practices and observations of MyFootCare with participants. Data were analyzed qualitatively through thematic analysis.

**Results:**

Key themes were as follows: (1) participants already used mobile phone photos to monitor their DFU progress; (2) participants had limited experience with using mobile phone apps; (3) participants desired the objective DFU size data provided by the tracking feature of MyFootCare to monitor their DFU progress; (4) participants were ambivalent about the MyFootCare goal image and diary features, commenting that these features were useful but also that it was unlikely that they would use them; and (5) participants desired to share their MyFootCare data with their clinicians to demonstrate engagement in self-care and to reflect on their progress.

**Conclusions:**

MyFootCare shows promising features to engage people in DFU self-care. Most notably, ulcer size data are useful to monitor progress and engage people. However, more work is needed to improve the usability and accuracy of MyFootCare, that is, by refining the process of taking and analyzing photos of DFUs and removing unnecessary features. These findings open the door for further work to develop a system that is easy to use and functions in everyday life conditions and to test it with people with DFUs and their carers.

## Introduction

Diabetic foot ulcers (DFUs) are common, costly, and take a toll on patients, families, and communities [1]. It is estimated that at any one time, DFUs affect between 3 million to 49 million people worldwide [1]. In Australia alone, each day, 50,000 people suffer from a DFU, 1000 are hospitalized, 12 have an amputation, and 4 die because of a DFU, leading to an estimated annual cost of Aus $1.6 billion [2,3].

DFUs often result from a combination of diabetes-related peripheral neuropathy (loss of protective sensation as well as changes in gait) and mechanical pressures (from walking or external trauma) [1]. When DFUs are complicated by peripheral artery disease and infection, they may take months or even years to heal and often lead to hospitalization, amputation, and even death [1]. In addition, DFUs impact the physical and mental quality of life of patients and their partners and families, with patients frequently describing a loss of independence over basic activities of living and a disruption to their sense of self as a result of the ulcer [4].

Best practice treatment of DFUs requires biweekly multidisciplinary team treatment in specialized clinics, with various clinicians working together to provide effective clinical care [5]. However, this treatment also relies on self-care away from the clinic: patients need to prevent excessive moisture, change wound dressings regularly, ensure cleanliness, moisturize, check their feet to identify changes in the wound and any potential infection, and, perhaps most importantly, adhere to wearing offloading devices at all times to relieve mechanical pressures and protect the ulcer [5]. These self-care practices are typically established in consultation between patients, carers, and multiple clinicians.

Unfortunately, adherence to self-care practices has been found to be typically low [6]. Patients often have a limited understanding of diabetes, foot ulcers, and the significance of self-care [7]. Furthermore, several studies have shown that knowledge alone is not enough for people to adhere to new practices [4,8]. Patients and their families also need to have the ability to enact care in terms of skills, time, finances, and resources [8,9]. In addition, and perhaps most importantly, patients need to be motivated to enact self-care consistently over months of DFU treatment [10]. Unfortunately, many patients view self-care practices as a further diminishment to their quality of life, such as wearing an offloading device at all times, while improvements to their ulcer when adhering to this care can be difficult to detect on a daily basis [4]. Hence, experts recommend that new strategies are needed to help motivate patients and engage them in self-care away from the clinic [6].

Mobile health apps hold great promise for people with diabetes, but few apps seek to engage people in their DFU self-care. A variety of apps for people with diabetes are available on the Google Play Store and the Apple App Store. These commercial apps provide health information or allow tracking of blood glucose levels, eating habits, and physical activity [11-13], but they do not target DFU care. Several apps are being developed to measure DFU size [14-17], recognize signs of infection [18], identify spots where new DFUs are likely to develop [19], and assess patients remotely [20], but these apps are targeted at clinicians rather than patients. A notable exception is the work by Boodoo and colleagues [21], who are working toward a DFU monitoring tool for patients. However, their tool relies on a near-infrared light attachment to the mobile phone, which limits accessibility for patients.

We recently developed a mobile app prototype called *MyFootCare*, designed for patients to motivate and engage them in their self-care [22]. MyFootCare encourages patients to use their own mobile phone to take photos of their feet. The app applies novel visual analytics to these photos to extract DFU size information that lets patients and their carers track their DFU healing progress [22]. Furthermore, MyFootCare highlights personal goals to help motivate patients and provides reminders to enact care on a regular basis [22]. The aim of this study was to evaluate the usability and potential usefulness for promoting self-care of an interactive prototype of MyFootCare with people with DFUs, based on a user-centered approach.

## Methods

### MyFootCare Prototype

The overall goal of MyFootCare is to be a mobile phone app that optimizes the engagement of people with DFUs in their self-care away from the clinic. MyFootCare was conceived by the research team based on their experience in the treatment and study of people with DFUs (JJvN and PAL) and in the design and implementation of mobile health technologies (BP and RB). The team developed multiple features within MyFootCare to engage people with DFUs, including the ability to visualize personal goals, self-monitor their DFU through ulcer photos and ulcer size information, a diary to foster reflection, and reminders to enact self-care [22].

The prototype presented in this study was the result of an iterative, user-centered design process. Multimedia Appendix 1 shows our initial prototype, which was implemented in Axure (Axure Software Solutions) [23], a prototyping software to generate interactive screen mock-ups to gather feedback from prospective users. On the basis of patient feedback, we refined the design and implemented a fully functioning Android app to demonstrate the feasibility of our approach. The Android app was based on Java frameworks and open source computer vision library (OpenCV) [24], a free real-time computer vision development library. A morphological watershed algorithm [25] provided by OpenCV was used to segment the foot from the image background and then the ulcer from the foot. The app relied on a small (1 cm diameter) green sticker on the foot to provide a scale for calculating the ulcer wound size [14]. The mobile phone flash was used to control lighting during image capture, that is, to illuminate the foot and keep the background dark. The prototype was developed and evaluated on a Samsung Galaxy S4 mobile phone.

The primary aim of this prototype and study was to demonstrate the feasibility of DFU monitoring to patients during an interview to obtain feedback on usability and potential usefulness. Hence, the following sections describe the features of the app and how participants in this study could interact with it during the interview.

#### Goal Image

**Figure 1:**
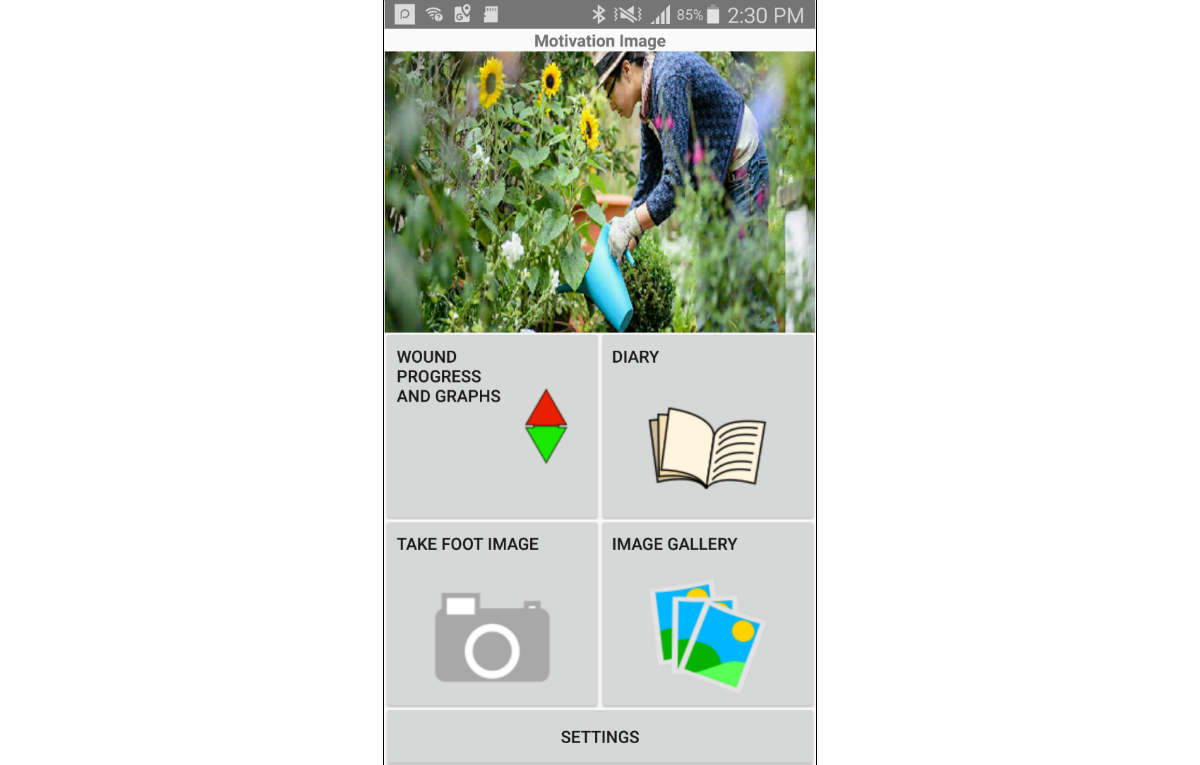
MyFootCare home screen showing a photographic image at the top to visualize a patient’s goal (eg, to enjoy gardening again) and access to all features.

The home screen (Figure 1) shows an image to visualize a goal a patient wishes to achieve when their DFU has healed. This feature was included because setting a realistic goal is typically one of the first steps in a therapy process to direct the treatment plan and to motivate patients to enact the plan [26,27]. The aim of this feature was not to quantify goals set with clinicians but to provide motivation. By having this image on the home screen, patients would be reminded each time they opened the app of their long-term goal of trying to achieve healing in a positive way.

Participants in this study could change the goal image by clicking on the image itself. They could choose from several photos provided in the app such as to enjoy gardening or to play with grandchildren. Alternatively, they could set a personal photo taken through the mobile phone camera or transferred from another device.

#### Capture Foot Photo and Analyze Ulcer Size

**Figure 2:**
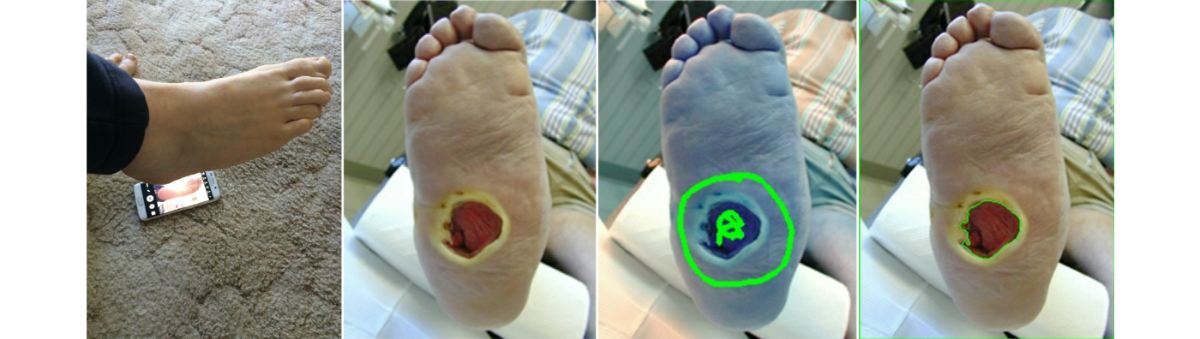
Photos can be captured with voice assistance. The analysis is based on circling around and inside the wound image to segment the ulcer from the foot.

Figure 2 shows the 2 steps involved in the feature capturing photos of the foot and analyzing ulcer size. First, patients need to take a photo of the whole foot. We expected that photos will usually be taken by a family member because even for healthy adults, it is difficult to take a photo of the plantar side of the foot.

For patients living on their own, we devised a voice assistance mechanism to help patients take photos without assistance from other people. People place the phone on the floor and hover their foot over the phone. The app guides the patient through voice feedback; specifically, the app vocalizes the phrases *higher* and *lower*. The guidance is based on image analysis through OpenCV. The app guides the patient to center the foot over the camera at an appropriate distance and then automatically takes a photo without the patient having to touch the phone. Finally, MyFootCare vocalizes *image successfully captured* to provide explicit feedback (Figure 2 leftmost image).

During the interview, the voice assistance feature was demonstrated by the researcher by hovering his foot over the phone and allowing the participants to hear the voice feedback to understand the concept. Although this feature was not accurate enough for patients to take photos themselves, we wanted to investigate if such voice assistance would be useful for patients.

Next, we developed a visual analytics feature (again based on OpenCV) to detect the ulcer and calculate its size. To evaluate this feature, participants used a test image that had been uploaded to the phone before the interview (as illustrated in Figure 2). To segment the ulcer and calculate its size, participants had to roughly draw on the image around the ulcer to denote skin tissue to the feature and then inside the ulcer using their finger on the screen of the phone to denote ulcer tissue (Figure 2 third image from left). The last image in Figure 2 (rightmost image) shows how the visual analytics feature then automatically segments the ulcer tissue from the foot image using an automated green line.

**Figure 3:**
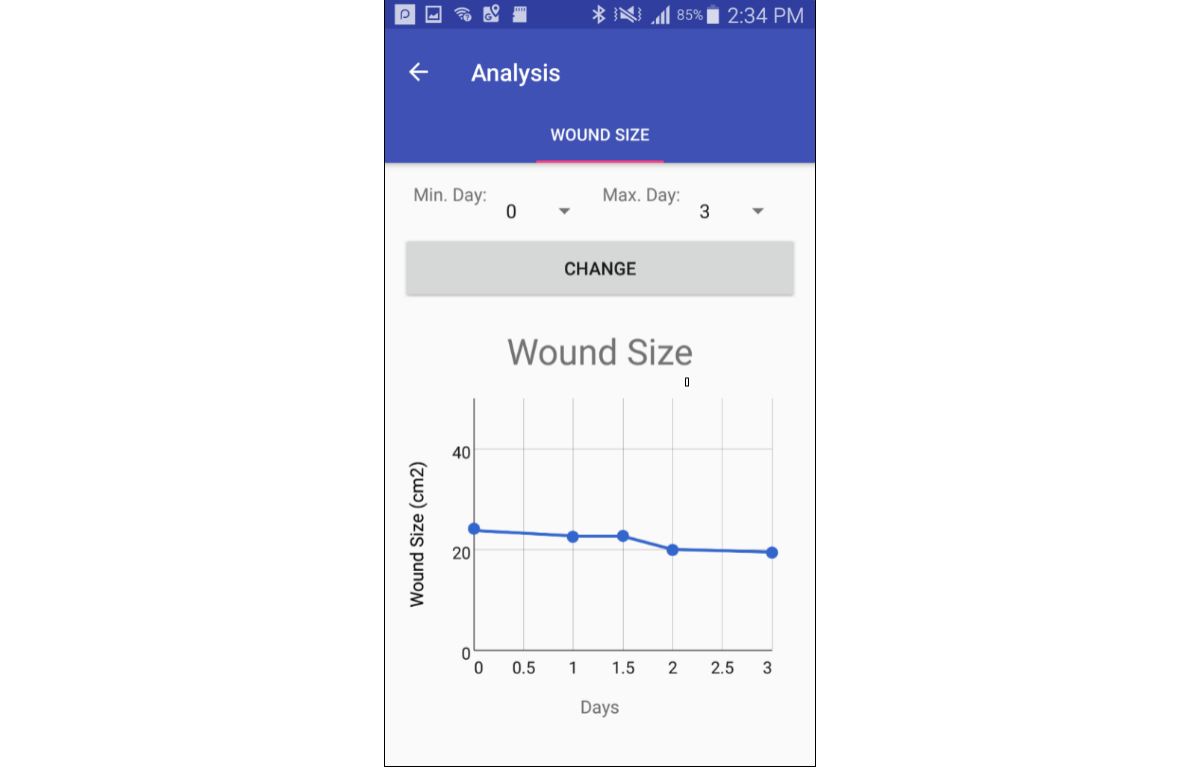
Patients can track the healing process in terms of wound size.

#### Wound Progress

On the basis of the ulcer detected in the image, MyFootCare calculates the size of the ulcer as a proportion of the size of the foot and presents the progress through a graph (Figure 3). Through this graph, patients can track their DFU healing process, which is often difficult to detect to the naked eye over weeks and months of the typical ulcer healing duration. This approach is inspired by popular self-tracking [28], quantified self [29] and personal informatics [30] approaches, which argue that personal health data can foster personal reflection and behavior change. Although it often takes a long time to heal ulcers, prior research suggests that the progress (or lack thereof) during the first 4 weeks provides a clear indication as to whether the ulcer care is effective (>50% reduction in ulcer area in the first 4 weeks of care has been found to be a surrogate marker of effective DFU healing [31-34]). Participants in this study could view the graph, which included the information generated by the researchers before the interview, as well as the information generated by the participants during their analysis of a test image.

#### Diary

**Figure 4:**
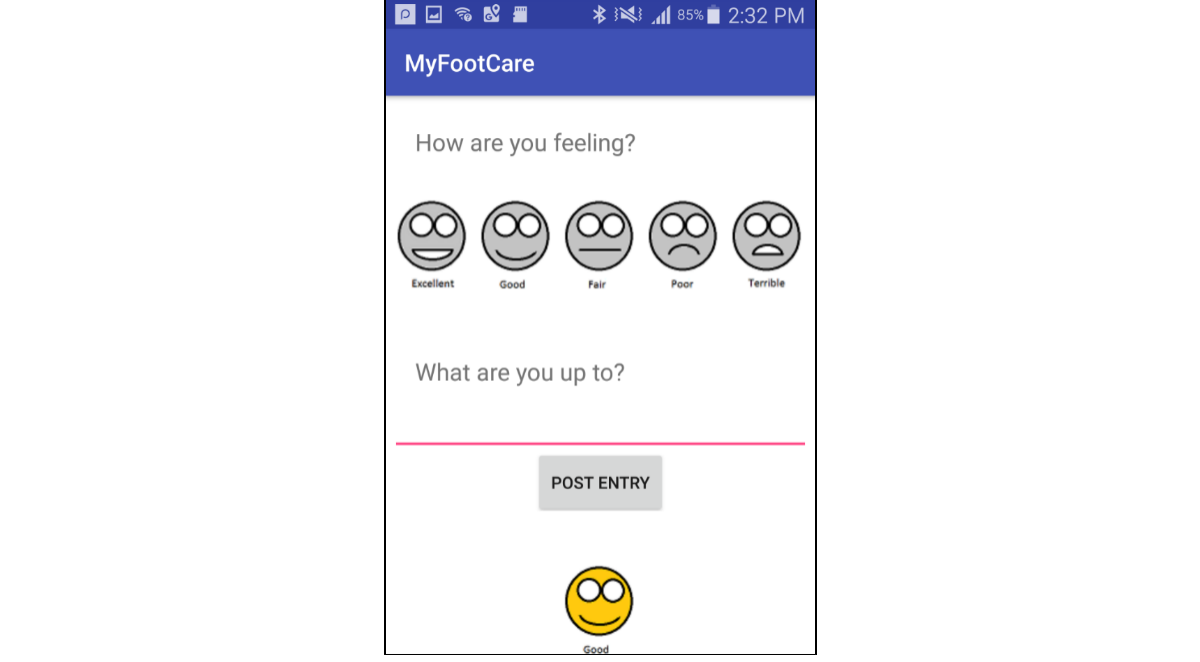
Patients can diarise information to reflect on their well-being and self-care.

The diary feature was incorporated to encourage reflection on self-care and well-being more broadly. Although we initially considered structured questions to help inform the therapy process, we eventually designed the diary in an open-ended manner so that patients can reflect on experiences that matter to them. Smiley faces were also added to let people add an entry quickly without having to type an entry (Figure 4). Participants were asked to add a diary entry during the interview and to comment on what information they would diarize, if any.

**Figure 5:**
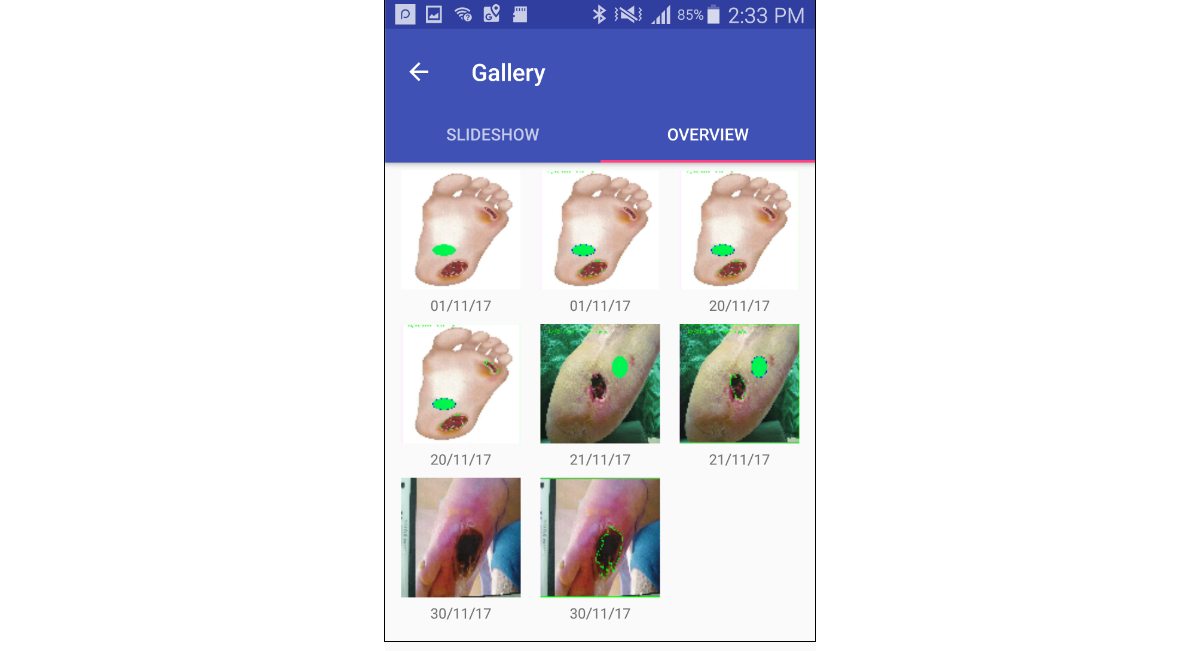
Image gallery allowing patients, carers, and clinicians to review ulcers visually.

#### Image Gallery

An image gallery allowed patients to review images and to see progress in the healing of their ulcer over time, in addition to the aforementioned graphing of progress. This image gallery was separated from the image gallery on the phone because patients may not want their ulcer images alongside other personal photos. We added the gallery feature to let patients revisit their images and also so that they can show their images to carers and clinicians (Figure 5). Participants in this study could browse through a gallery that contained sample images provided by the research team.

#### Reminder Notifications

**Figure 6:**
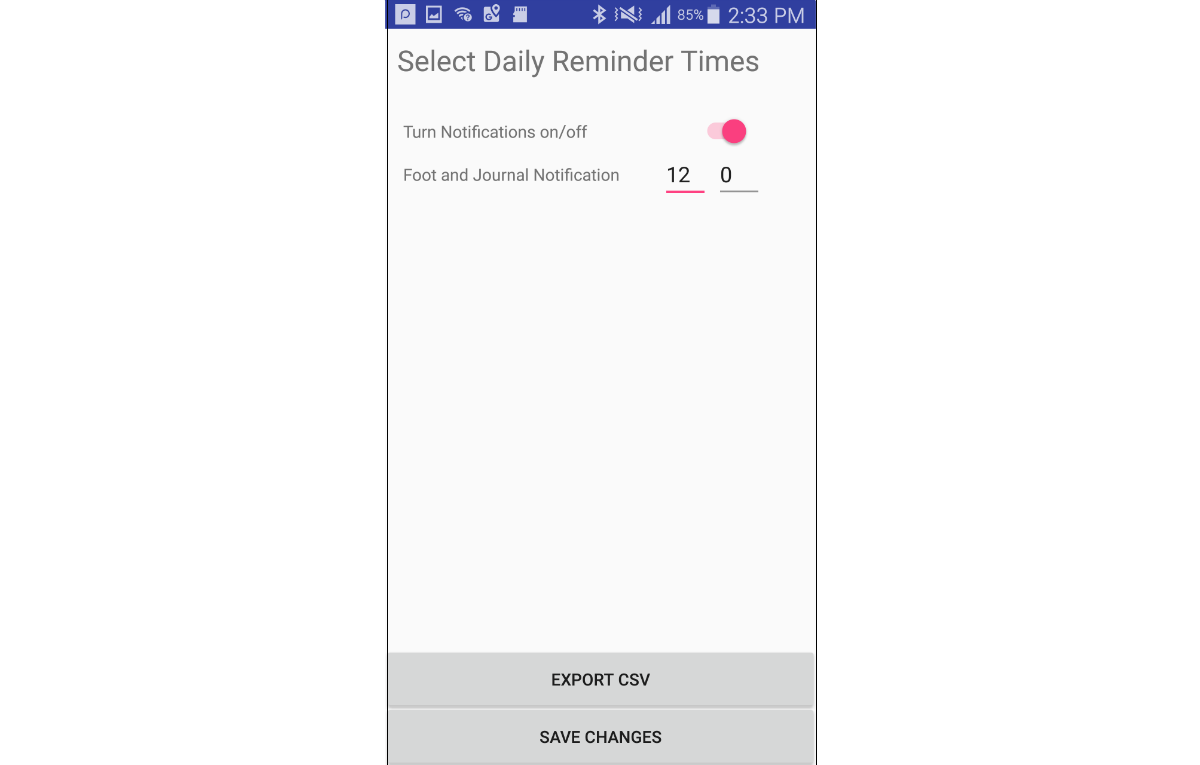
App reminder configuration interface.

The app also provides patients with notifications to remind them to enact their dressing changes, take ulcer photos, or to make an appointment with their clinician. We added this feature because behavior change theory [10] suggests that even if people have sufficient knowledge and motivation, they may forget or run out of time and therefore need a reminder to enact behaviors. Reminders are implemented using a simple dialogue under settings, defining the intervals for reminding the patient to take photos and use features in the app (Figure 6).

Participants were asked to set the time for notifications, which prompted discussion about the potential usefulness of notifications and its contents. Furthermore, participants could view a sample notification on the Android lock screen that stated *Time to check your foot*, which they could double-tap to open MyFootCare.

### Study Participants

Eligible participants were people with a DFU being treated at a diabetic foot clinic and who owned a mobile phone. DFUs were defined as a full-thickness wound on the foot (ie, below the malleoli) of a person with diagnosed type 1 or type 2 diabetes mellitus [1]. An internet-enabled mobile phone was a requirement so that participants would have some familiarity with mobile phone apps and potentially be willing to use it on their own phones. Recruitment was conducted through a large community diabetic foot clinic in Brisbane, Australia.

In all, 11 participants took part in this study (10 men and 1 woman who were aged between 43 and 74 years). All participants had had foot ulcers for extended periods, ranging from 3 months (P11) to recurring ulcers for 7 years (P5). All 10 male participants (P1-10) had a spouse or child who helped them care for their ulcer, whereas participant 11 looked after her own ulcer. The carer of participant 4 also joined the interview to provide an additional perspective. All participants owned mobile phones, but only 6 of them regularly used apps on their phone (P1, 3, 4, 5, 6, and 8).

### Data Collection

We conducted a qualitative evaluation through semistructured interviews to explore how people with DFUs would use MyFootCare and to what extent the app could enhance their self-care practices. The interviews took place in a meeting room at the clinic where participants received their foot care and lasted 30 to 60 min per participant. Ethics approval was obtained from The Prince Charles Hospital’s human research ethics committee (#17/QPCH/14).

The evaluation followed a standard procedure. First, a background interview was conducted to learn about their ulcer history, clinical care and self-care practices, and mobile phone usage. Second, we conducted observations of patients exploring each of the MyFootCare features. The participants were given a mobile phone with the MyFootCare prototype. They were instructed to think aloud to get a better understanding about their impressions of each feature, any questions or expectations that they may have, and whether they would try out this feature on their own phone. Participants were free to try features in any order they wished, and questions were asked accordingly. Finally, through a semistructured interview, the participants were asked to compare and rate the features in terms of usefulness for their DFU care. These ratings were used as prompts to discuss how the app could be integrated with their self-care practices and the potential impact on improving their therapy process. Each evaluation was conducted by the same researcher (LSDS) and was audio-recorded and transcribed verbatim for later analysis.

### Data Analysis

The data were analyzed qualitatively, following a thematic analysis approach [35]. The authors read through all transcripts and coded the data to identify the various uses for each app feature as well as areas for improvement. Data were coded by the authors (BP, JJvN, and LSDS) through SaturateApp, a Web-based tool for collaborative qualitative analysis [36]. In total, 54 codes were generated about the existing mobile phone practices, 97 codes about MyFootCare features, and 57 codes about the potential use in daily life. These codes were collated into 5 themes that describe existing care and mobile phone practices and how MyFootCare could support them, and they are presented in the Results section.

## Results

### Theme 1: Participants Already Use Mobile Phone Photos to Monitor Diabetic Foot Ulcer Progress

Mobile phones were already an integral part of self-care for many participants. Overall, 8 out of the 11 participants had photos of their ulcers on their mobile phone. This suggests that MyFootCare can build on well-established practices among people with DFUs.

The main motivation for participants to take these photos was to monitor progress. Participants found progress difficult to assess on a day-to-day basis for several reasons: they could not feel the ulcer because of peripheral neuropathy and participants also found it difficult to see their ulcer by themselves as their ulcers were on the plantar side of their foot and typically covered by dressings or a cast. Hence, participants often relied on others to take a photo at times of wound dressing changes:

I get the wife to take the photos. When they were changing the cast at the hospital I’d ask the nurse to take a photo so I could see what state my foot was in.

P9

More importantly, healing takes weeks or months, and hence, improvements are difficult to ascertain without a record, as pointed out by participant 7:

See the thing is with wound care you just, I can’t tell the difference; you see it every day you might not notice the changes.

P7

Progress information from photos was important for participants as well as their carers (most often their partners):

I’ve quite often scrolled back through the photos looking for an older one just to, so that I have a visual comparison.

Carer of P4

Some participants found seeing progress in photos encouraging:

No one wants to see a photo of a chronic ulcer, but for me it shows where I’ve come from, what it looked like then, and what it looks like now.

P3

Those participants who did not have photos of their ulcer on their phones (P5, 7, and 11) received photos from their podiatrist to check their progress. For example, participant 5 stated the following:

I do that every week when I come here [to the clinic]. They normally take a photo and then I can see it.

P5

This highlights that all participants in this study were already relying on photos to monitor their progress.

However, we also found that ulcer photos were not taken in a systematic manner. Participants had only a few photos on their phones, although they had their ulcers for several months or even years. Photos appeared to have been taken in an ad-hoc manner at different angles, distances, and periods, rather than in a systematic way. The photos of participant 9 did not have the correct dates because they were taken by his wife and children on their own phones:

Mum sent a copy because she wasn’t able to get up to the hospital with me, my son sent one, my daughter sent one and there’s so many copies in there, they’re all out of sequence.

P9

### Theme 2: Participants Have Limited Experience With Using Mobile Phone Apps

The widespread use of ulcer photos was encouraging, particularly because only 6 out of the 11 participants regularly used apps on their mobile phones. The remaining 5 participants stated that they used their mobile phone only to call other people and occasionally to send and receive short message service text messages. Participant 7 stated that he was “not a smartphone person.” Some participants commented that they were too old. For example, participant 10, a 53-year-old man, commented the following:

I just haven’t bothered with any of it; it’s my age, I just don’t [use apps].

P10

Participants also highlighted issues that limited their ability to access and use mobile phone apps in general. One difficulty was limited dexterity, which makes navigating and typing on a mobile phone cumbersome. For example, participant 2, a 74-year-old man, stated the following:

The problem I have is my hands, my dexterity’s not that good [...] for me to type in the stuff it would take me 20 minutes or half an hour.

P2

Furthermore, participants reported difficulties reading on mobile phones, which is not surprising considering people with diabetes often also develop diabetes-related complications of retinopathy and blindness. For instance, participant 9 stated the following:

...that’s too small an interface for my eyes because I’ve had retinopathy, I’ve had laser surgery on both eyes, I’ve had cataracts removed off both eyes.

P9

### Theme 3: Participants Desire Objective Data From MyFootCare to Monitor Diabetic Foot Ulcer Progress

Feedback regarding MyFootCare was largely positive. Overall, 7 out of 11 participants said that they would be interested to try out the app on their own phones for several weeks to support their self-care.

The key benefit of MyFootCare for participants was that the app could provide objective data to monitor the progress of their ulcers. Participants could clearly see how they could monitor progress by taking photos on a regular basis and by tracking the objective ulcer size information provided by the visual analytics feature. The participants highlighted that MyFootCare would make ulcer size more explicit:

It’s so handy especially if you’ve got no idea. In my case I don’t see a lot of the wound so knowing the size is handy because then I can tell whether it’s actually a problem or becoming more of a problem than you know just going along and all of a sudden, and I’ve done it before, going along well and all of a sudden my wound’s fifteen by three or something, which is not ideal.

P5

Seeing progress through the app is particularly important because ulcers often heal slowly. Hence, participants often felt demotivated by the lack of visible progress, which they hope would be addressed by being able to track ulcer size over several weeks or months with MyFootCare:

If I took a photo of something every day I’d get frustrated ’cause look now, it’s not changed. But if you do it a week apart, you just have to [see change].

P7

The desire for seeing progress and the potential motivation to keep up good self-care was highlighted several times:

Just proving to yourself that the ulcer is getting better.

P11

You can see the progress; and when you can see progress you’re more inclined to keep doing the right thing.

P9

Importantly, participants regarded the data on MyFootCare as objective data, independent from their own subjective well-being, as highlighted by participant 1:

It’s not going to lie. It’s going to ask the same questions each time and it’s going to be yes/no answer basically. Is it bigger? No, it’s not. Is it smaller? Yes, it is.

P1

It is also important to note the limitations pointed out by participants. First, participants recognized that taking photos of the plantar side of their foot to provide such objective data may be difficult, but that the automated image taking feature contained in MyFootCare may provide a solution to this difficulty. Images need to be consistently taken at a certain angle and at a certain distance to provide accurate data:

I’d say with certain parameters within [the app] that recognises that OK you’re holding it at this angle or that angle and that’s why it’s saying no take the photo again. Or you know it’s supposed to be between ten and fifteen centimetres or what have you so it can do all the calculations.

P1

Although the researcher could demonstrate the image-taking process during the interview, participants and their carers noted that taking an image at home might be difficult and that assistance from another person might be needed:

It’s probably not so much a case of [P4] taking the photos himself but one of us doing it for him because yeah it’s too hard to manoeuvre with one hand.

Carer of P4

Second, not all participants were interested in trying out MyFootCare. As discussed above, participants 2, 7, and 10 stated that they did not use any mobile phone apps and hence would not use MyFootCare either. Participants 5 and 10 felt that their ulcers were healing well and said that they did not see the need for additional support through an app:

If they got bad yeah, I could see it; but because we’re onto it straight away I really haven’t had a problem.

P10

Participant 7 stated that he did not see the need for MyFootCare because clinicians were already taking photos for him:

Every two weeks they take a photo and they can, that’s all on file, well you know the folder. And you go back all this time you can see what my foot was doing a year ago, what it was doing six years ago, six months ago, what it was doing six weeks ago.

P7

### Theme 4: Participants Were Ambivalent About the MyFootCare Goal Image and Diary Features

Participants felt ambivalent about the goal image and diary features. They could see the potential benefit of using these features to find motivation and to reflect on factors that may influence their self-care and their progress. At the same time, however, many participants stated that it was unlikely that they would use these features in daily life.

Goal setting is an integral part of any therapy. However, the feedback on the MyFootCare feature to set an image that represents their goal was mixed. Participant 7 highlighted that goals are important to stay motivated to look after the foot:

You definitely need motivation; You’re going through these emotional ups, lows and that really, no that’s, motivation is always good.

P7

Discussing this feature with participants has also highlighted the various goals that they were pursuing. The main priority for most participants was for the ulcer to heal or to avoid amputation:

I want to heal the ulcer in the shortest possible time, I don’t want to have to wear medical grade footwear, I don’t want to have to wear a crow boot. My motivation is to have the problem resolved in five or six months for argument's sake. Some of us might put unrealistic expectations on that and if we don’t get it done. Yeah but my motivation comes down to I want to live a long life with my legs. I don’t want to lose them.

P9

Participants also highlighted activities that were important for them and motivated them to get their ulcer healed, such as being able to shower, engaging in physical activity, and playing with their children:

I would like to go swimming with my kids and not have to worry about the foot getting wet or the bandage getting wet.

P8

Moreover, 6 of the participants also highlighted that (unlike the ability to monitor progress) having an image on the app is not essential to the app. They stated that they were aware of their goals and did not need them visualized:

Having progress is probably more important, giving an idea of where you’re going. But I don’t know that motivation, I think most people try and be motivated by some form so I don’t know that that’s a huge thing.

P2

The diary also received mixed feedback. Some participants (P1, 3, 4, 7, 9, and 10) pointed out that it provides a useful feature to reflect on contextual factors that might impact progress. Participant 1 recognized that the diary can provide context to the ulcer measurements (as provided by the visual analytics feature) and that it can aid personal reflection on factors that influence healing:

You know that’s a diary, you put in comments that you want to, you might get “OK ulcer grew this week but decided to go for a walk around IKEA.” So you know like you know that you did have a problem but you’re also putting sort of like the reason why. And so you can sort of possibly learn the things to avoid and what have you, how to adapt your lifestyle for better healing so to speak.

P1

Participant 9 indicated the potential value of the diary to aid reflection during consultations with podiatrists:

I come along to you to get my foot done and you’re saying what did you do, well I can’t remember, look up my diary.

P9

Despite recognizing these benefits, participants stated that it would be unlikely that they use the diary. Participant 6 stated the following:

Well it’s not a bad idea with the journal but I probably wouldn’t use it myself.

P6

Participants mentioned that it would require effort:

The diary is good providing you do it every time [...] it can be a bit laborious.

P2

Furthermore, the personal benefit of the diary was not clear to participants:

From my point of view I don’t see that as an advantage, probably might be for the healthcare worker.

P11

### Theme 5: Participants Desire to Share MyFootCare Data With Their Clinicians

Overall, 9 out of 11 participants pointed out that MyFootCare data would be useful for discussion with their clinician. Although this was not an explicit feature of the app, participants suggested that the information available through MyFootCare would be useful for consideration during consultations with their podiatrists and general practitioners (GPs):

That would be good because then I could show my doctor and say look this is the progress we’re having. If I see another podiatrist, I mean I know it’s in my file, but it’s a nice easy way for them to look at it and go hey look OK right-o!

P3

The photos, progress charts, and diary information have the potential to provide clinicians with information about the participant’s well-being in their everyday life environments:

It would give the podiatrist a better feel of what’s going on I think. They see what’s happening at home, they see what’s happening when you’re not here [in the clinic]. You get to see them for ten/fifteen minutes, there’s not a lot of time. And because that’s because there are so many people with this problem. So that would give them a weekly feedback on what’s been happening during the week, how your toes have been looking or your ulcers are looking when you’ve been changing the dressing.

P3

Furthermore, 5 participants also expressed a desire to digitally share MyFootCare data with a clinician outside of consultations. Participants pointed out that sharing information from MyFootCare would allow them to keep their GPs and podiatrists up-to-date with their progress in between consultations:

With your health care provider being able to send [to] them, let them know the sizes or the images, that's very important.

P8

In addition, participants pointed out that they would like to share MyFootCare with a clinician to determine if they need to see them in response to a deterioration of their DFU. For example, participant 3 suggested adding a feature to contact a clinician for advice based on the photos and graphs:

A section, like a messenger, where you get online help if you’ve got a question; for example, “I noticed a different colour ooze coming out of the wound.” You can share the photo and ask, “should I contact my podiatrist or can it wait to the next appointment?”

P3

Participant 1 said that used this way, MyFootCare would allow patients and clinicians to be more proactively engaged in their care:

It’s being nearly proactive rather than reactive.

P1

## Discussion

### Principal Findings

This study showed that people with DFUs perceive a mobile phone app such as MyFootCare as useful to engage them in the care of their ulcers. Despite technological advancements and despite the burden of the complication, mobile phone apps are hardly used by patients in their management or prevention of DFUs. Some pilot research in this area focused on mobile phone apps that use thermal cameras attached to mobile phones to detect signs of possible ulcers early on [19] or to manage active ulcers [21]. Unlike these apps, however, our design works with standard mobile phone cameras, which makes it potentially available to anyone owning such a mobile phone without further cost or work. In addition, other apps are being developed mainly to measure DFU size [14-17], but these apps are targeted at clinicians treating patients rather than patients engaging in their own care. Our app differs by being patient-focused, including a patient-oriented design, involving patients from the start of the research, and aiming to improve patients’ motivation by developing an app for them to use rather than keeping the app in the hands of the clinician.

Patients perceived the main benefit of MyFootCare was its visual analytics feature that provides objective data about the size of ulcers from photos of the foot. This information was seen as valuable because patients typically cannot feel or see their ulcer, and even if they could see their ulcer (on photos or in person), they could not detect if it was improving or deteriorating. In addition, the participants regarded the information provided by the app as objective and hence put more faith in this information than in their own or their carer’s subjective accounts. Importantly, the app may address a lack of motivation by patients by showing them progress in their healing process [10]. This may encourage patients and their carers to continue self-care practices in a consistent manner.

Many patients in our study already used mobile phone photos (mostly taken by others) to inspect their ulcers. Although related work shows that people with diabetes take photos of the food they have eaten and share them with dieticians [37,38], our study now shows that many people have also already adopted mobile phones to take images of the foot to share with relevant others (either clinicians or carers). This also makes it more likely that people will use MyFootCare to take photos and track their healing process in real life.

Although feedback on MyFootCare was largely positive, we also identified several challenges. First, using an app does constitute additional work for the patient and thereby increases the already significant workload involved in their ulcer care and diabetes management. Monitoring progress was seen as valuable, but participants also indicated that further reflection through goals and diaries might not be worthwhile enough to warrant the extra work. Goal images were included because reflection on goals and progress data can be empowering, as it helps explain the relationship to people of how their care activities can impact their progress and ultimately their goal [39]. Writing a diary was included as it can help people to come to terms with difficult issues [40] such as the disruption caused by a DFU. However, the participants in this study were ambivalent about the goal and diary features. They could see potential benefits, for example, by providing more detailed information to their clinicians, yet they also felt that the effort would not be justified by these benefits. In future iterations of MyFootCare, we recommend to potentially remove these features and keep the focus on self-tracking.

Second, many people with DFUs find mobile phone apps difficult to use. Although we recruited only mobile phone owners, many of them did not use apps on their phone, and some participants found apps inaccessible because of limited vision and dexterity. This finding is consistent with other studies of mobile phone apps for people with diabetes. Despite increasing availability of diabetes apps, they are often not well designed to support the needs of people with diabetes, that is, for older adults [11-13]. In moving forward in the development of the app, we will continue to explore further guidance in the image capture process, for example, through voice assistance mechanisms or selfie sticks to control distance and lighting between phone and the foot, through boxes to rest the foot for image capture [16], or through consistent *ghost* outlines of the foot on the camera screen each time an image is taken to keep photos consistent in angle and distance [17]. We will also explore the use of tablet computers, which may provide a better grip and a larger surface area to make the app more accessible for people with limited vision and dexterity. In exploring these options, it is important to continue working with people with DFU of all ages and their carers to ensure that the design allows them to easily read and navigate information.

Finally, we found that many participants wished to share their data with their clinicians. This is both a challenge and an opportunity. On one hand, the desire to share information aligns with growing trends in digital and participatory health care [41,42], where patients increasingly take control of their own health and related information. Photos are particularly popular in participatory health care approaches because they are easy to generate and interpret [43-45]. At the same time, however, sharing information with clinicians creates challenges in terms of information ownership, security, and privacy [46]. It also raises the question of feasibility, with previous studies highlighting that mobile phone images of DFUs in isolation may not be sufficient for clinicians to make reliable diagnosis [20]. Furthermore, it would also require a change in organizational practices, where clinicians receive time and remuneration for reviewing such information without the presence of patients. To overcome barriers to sharing data electronically, we recommend patients keep their data on their own mobile phones. Patients can then choose what data they show to clinicians during consultations, which avoids technical and legal pitfalls and allows patients to remain in control of their data.

### Limitations

First, the findings from this study come from a small cohort and may not be representative of all patients with DFUs. We only recruited patients who already owned mobile phones, and still, some patients within our cohort did not use apps at all. During our recruitment phase, we found that many patients did not have mobile phones, which is supported by survey studies showing that mobile phone ownership among individuals with diabetes is lower than that in the general population, especially among older adults and people with low incomes [47]. Although this may change in the future, it is important to note that the encouraging findings presented here do not reflect the opinions of all DFU patients.

Second, the accuracy of MyFootCare has not been evaluated. The aim of this app prototype was to demonstrate the feasibility of monitoring DFUs to patients to obtain feedback on the potential usefulness for self-care. Now that we understand that patients desire objective data from MyFootCare to monitor DFU progress, our future research will focus on iteratively evaluating and improving the accuracy of the app. Evaluations will be performed by comparing MyFootCare measurements with measurements performed by clinicians using ruler measurements [14], wound tracings [16], or gold standard digital wound imaging instruments [48,49]. Accuracy will be improved by working with patients to assist them in controlling factors such as light, distance, and angle of the foot during image capture. We will continue to refine the voice assistance and also explore alternatives, for example, selfie sticks, light boxes [16], and *ghost* outlines of the foot [17].

A third limitation of this study lies in the ecological validity. The findings from this interview study provide rich insights into the potential uses of a mobile phone app to support self-care in people with DFU. However, they only express opinions on potential use based on trying out the app with the assistance of a researcher. Such evaluations of technology prototypes through interviews are an important step in a user-centered design process and commonly reported in the health informatics literature, including in the area of diabetes (eg, [21,50-53]). A critical next step is a deployment study where patients and their carers can use and evaluate the app over several weeks or months in their daily lives to quantify app engagement and to evaluate the actual impact on self-care.

### Conclusions

MyFootCare, a mobile phone app that seeks to engage patients through goals, progress monitoring, and reminders, shows promising features to engage people in DFU self-care. The patients in this study expressed positive views on MyFootCare. The features perceived most useful were (1) taking photos of foot ulcers to assess healing and (2) the ability to monitor changes in the size of their ulcers through wound size data generated from such photos. More work is needed to improve the usability and accuracy of MyFootCare, that is, by refining the process of taking and analyzing wound photos. This study enhances our understanding of opportunities and challenges for mobile health technologies, especially through medical photography, to support people with diabetes and DFUs. The findings open the door for further work to develop an app that is accurate, reliable, and easy to use in daily life and to test it with people with DFUs and their carers. The app presented in this study works on standard mobile phones without requiring a separate camera. With mobile phones becoming more widely used among people with diabetes, MyFootCare has the potential for widespread impact.

## Appendix

Multimedia Appendix 1 MyFootCare Axure prototype.

## Abbreviations

DFU: diabetic foot ulcer
GP: general practitioner
OpenCV: open source computer vision library
