# Supplementary material for: Promoting Self-Care of Diabetic Foot Ulcers Through a Mobile Phone App: User-Centered Design and Evaluation
Source: JMIR Diabetes. 2018 Oct 10;3(4):e10105. doi: 10.2196/10105 (PMC6238831; doi:10.2196/10105)
Supplement: Multimedia Appendix 1 [file diabetes_v3i4e10105_app1.pdf]

Multimedia Appendix 1: MyFootCare Axure Prototype

The Axure prototype can be accessed via https://7ma9b5.axshare.com/footcare.html with the access code: footcare

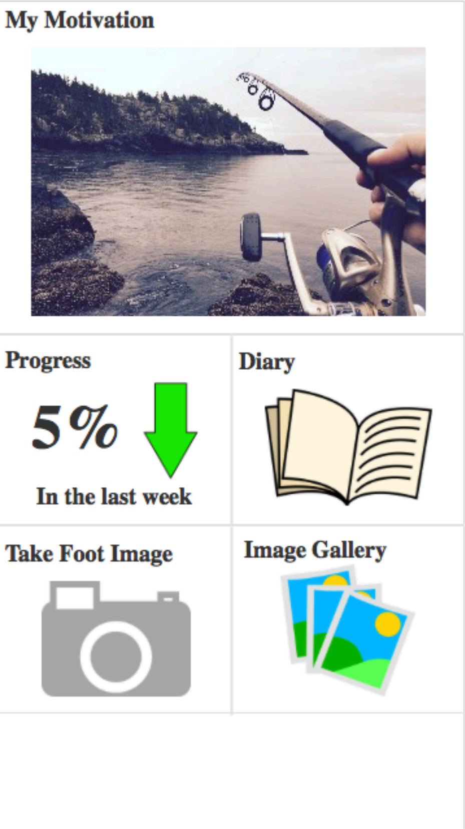

Figure 1: MyFootCare home screen, showing a photographic image on the top to visualize a person's goal (e.g., to walk in the forest) and access to all features.

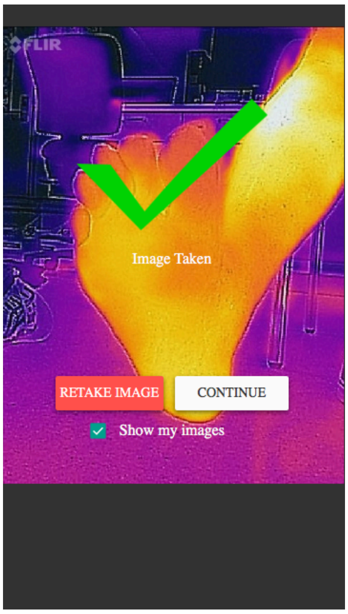

Figure 2: Take foot image and analyse ulcer. We considered thermal images from a Flir camera attachment for the initial prototype. However, the Android app prototype worked without thermal images to keep the app simple for patients.

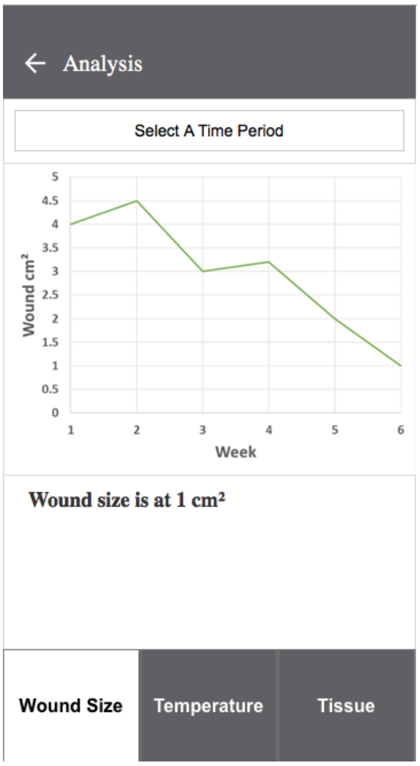

Figure 3: Patients can track the healing process in terms of wound size. Wound temperature and tissue analysis have not been implemented in the Android app to keep the app simple for patients.

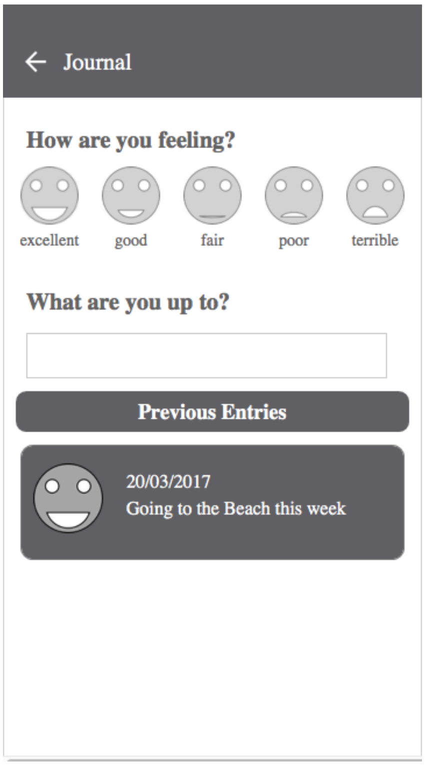

Figure 4: Patients can diarise information to reflect on their wellbeing and self-care.

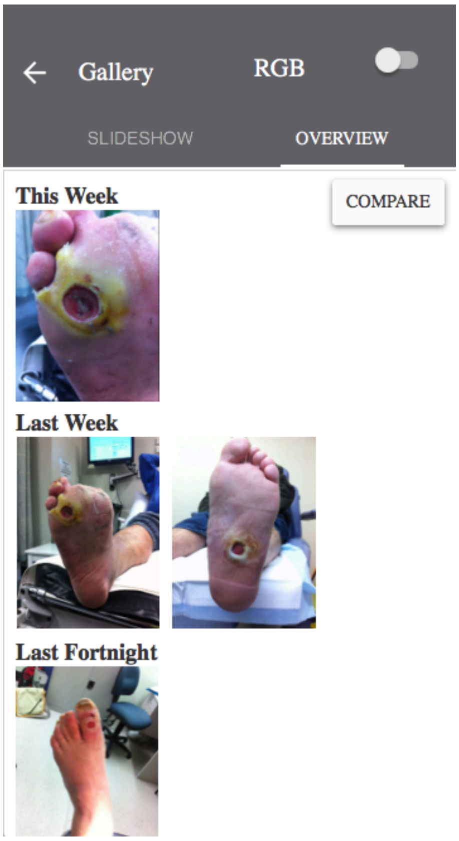

Figure 5: Image gallery allowing patients, carers and clinicians to review ulcers visually.
